# The Status of *Fusarium* Mycotoxins in Sub-Saharan Africa: A Review of Emerging Trends and Post-Harvest Mitigation Strategies towards Food Control

**DOI:** 10.3390/toxins9010019

**Published:** 2017-01-05

**Authors:** Cynthia Adaku Chilaka, Marthe De Boevre, Olusegun Oladimeji Atanda, Sarah De Saeger

**Affiliations:** 1 Laboratory of Food Analysis, Department of Bioanalysis, Faculty of Pharmaceutical Sciences, Ghent University, Ottergemsesteenweg 460, Ghent 9000, Belgium; Marthe.Deboevre@UGent.be (M.D.B.); Sarah.Desaeger@UGent.be (S.D.S.); 2 Department of Food Science and Technology, College of Applied Food Science and Tourism, Michael Okpara University of Agriculture, Umuahia-Ikot Ekpene Road, Umudike, Umuahia PMB 7267, Abia State, Nigeria; 3 Department of Biological Sciences, McPherson University, KM 96 Lagos-Ibadan Expressway, 110117 Seriki Sotayo, Ogun State, Nigeria; olusegunatanda@yahoo.co.uk

**Keywords:** *Fusarium* mycotoxins, modified mycotoxins, occurrence, food processing, sub-Saharan Africa

## Abstract

*Fusarium* fungi are common plant pathogens causing several plant diseases. The presence of these molds in plants exposes crops to toxic secondary metabolites called *Fusarium* mycotoxins. The most studied *Fusarium* mycotoxins include fumonisins, zearalenone, and trichothecenes. Studies have highlighted the economic impact of mycotoxins produced by *Fusarium*. These arrays of toxins have been implicated as the causal agents of wide varieties of toxic health effects in humans and animals ranging from acute to chronic. Global surveillance of *Fusarium* mycotoxins has recorded significant progress in its control; however, little attention has been paid to *Fusarium* mycotoxins in sub-Saharan Africa, thus translating to limited occurrence data. In addition, legislative regulation is virtually non-existent. The emergence of modified *Fusarium* mycotoxins, which may contribute to additional toxic effects, worsens an already precarious situation. This review highlights the status of *Fusarium* mycotoxins in sub-Saharan Africa, the possible food processing mitigation strategies, as well as future perspectives.

## 1. Introduction

*Fusarium* is one of the most important filamentous pathogenic mold genera widely distributed around the world. These molds are often referred to as field or soil fungi because of their great pathogenic potential, thus causing a wide range of plant diseases called fusariosis, such as vascular wilts, seedling blights, rots, and cankers [1,2]. Fusariosis causes enormous economic losses to crops thereby affecting trade and marketing worldwide. This is evidenced by the estimated crop yield reduction between 10% and 40% reported by Bottalico and Perrone [3]. In the USA, the genus *Fusarium* has been estimated to cause losses worth 2900 million US dollars annually for wheat and barley [4]. In addition to their pathogenicity to plants, *Fusarium* species are capable of synthesizing a wide range of secondary metabolites of diverse structures and actions. Species such as *F. verticillioides* and *F. graminearum*, each have the ability to synthesize more than one metabolite. *Fusarium* metabolites of economic importance include fumonisins, zearalenone, and trichothecenes. Their importance is partly ascribed to the presence of some base-line scientific data as well as documented significant impact on public health and animal productivity across several countries. These toxins have been implicated as causing several devastating diseases in humans and animals ranging from acute to chronic with carcinogenic, estrogenic, mutagenic, hepatotoxic, teratogenic, hemorrhagic, neurotoxic, and/or immunosuppressive effects [5]. They may co-exist in feeds, foods, and processed food products because some fungi have the ability to produce more than one mycotoxin, and/or more than one fungi species may colonize a substrate. Thus, an intrinsic quality is the exhibition of a synergistic, additive, and/or antagonist health effect on the human or animal host [6]. In addition to their harmful significances to health, mycotoxins are major food contaminants affecting global food security, especially in the developing countries. The Food and Agriculture Organization (FAO) of the United Nations estimates that about 25% of world food crops are contaminated with mycotoxins [7]. Cases of food destruction owing to high mycotoxin levels leading to losses of millions of dollars have also been reported [8,9]. Wu [10] reported an estimated annual economic loss of between 1 and 46 million US dollars as a result of fumonisin contamination in animal feed, leading to market and animal life losses in the United States. Losses resulting from all mycotoxin-related issues in agriculture in the United States have been estimated to be as high as 1.4 billion dollars annually [11].

The emergence and occurrence of new *Fusarium* metabolites in food crops and products is of great concern. The occurrences of emerging mycotoxins produced by *Fusarium* spp., such as fusaproliferin, beauvericin, enniatins, and moniliformin have been reported in food crops representing an important problem in some parts of the world [12,13]. The risk of human and animal exposure to these mycotoxins has led to continuing elucidation of chemical structures and possible further alteration of a *Fusarium* toxin's structure in crops and food products. Gareis et al. [14] observed some cases of mycotoxicosis symptoms in animals which did not correlate with the corresponding low-mycotoxin-contaminated feeds they were consuming. The elevated toxicity was ascribed to undetected conjugated forms of mycotoxins that were possibly hydrolyzed into the free toxins in the digestive tract of the animals. This is supported by the recent in vitro study of Gratz et al. [15] and Ajandouz et al. [16] which revealed the potential hydrolyses of conjugated mycotoxins into parent mycotoxins by the microbiota in the human gut. These undetected conjugated mycotoxins, referred to as modified mycotoxins, may be matrix-associated; biologically modified by plants, animals, or fungi; or chemically modified by thermal or non-thermal processing [17]. Recently, much attention has been channeled to modified mycotoxins, especially in the developed countries. Several studies have proven the existence of modified mycotoxins in crops and food products [18,19,20,21,22,23,24,25,26,27,28]. Conversely, the limited existence of toxicological data on modified mycotoxins has contributed to the difficulty in ascertaining their toxicity effects.

Although *Fusarium* mycotoxins have been associated with temperate climate, recent trends in climatic change seem to have exposed the tropics to these toxins. Magan et al. [29], and Paterson and Lima [30] emphasised the importance of climate in fungal colonization, as well as mycotoxin contamination of foods and food products. Unfortunately, sub-Saharan Africa (SSA) has been reported as a region of higher vulnerability to the impact of global climate change because of its sole dependence on the weather and climate variables for agricultural production [31]. SSA is envisaged to become 5% to 8% more arid and semi-arid, which perhaps will cause an increase in drought, and thus may lead to increased crop stress and possibly mycotoxin contamination [32]. Tirado et al. [33] highlighted the correlation between fumonisin occurrence and drought stress, as observed in maize planted during the dry season in South and Eastern Africa. The trend of increase in fumonisin production in dry weather was also reported by Munkvold and Desjardins [34].

Another possible route of exposure to *Fusarium* mycotoxins in SSA is through trade. Fungi can easily spread from one area to another, and considering that there are no strict regulations and control systems concerning mycotoxins in this region, SSA often times is exposed to contaminated foods and products through global trade. Studies have reported a high incidence of *Fusarium* mycotoxins in crops and food products in SSA [35,36,37,38,39,40]. A Biomin survey on the global mycotoxin threat also reported high incidences of zearalenone (91%) and fumonisins (88%) in a majority of samples from Africa [41]. Despite the increasing concern on *Fusarium* mycotoxins and their modified forms worldwide, SSA has placed little importance on the occurrence and detection of these mycotoxins in crops and food products, as well as their possible deleterious effects. Perhaps this could be as a result of the non-availability of analytical facilities and the prevalence of food insecurity in the region. Another school of thought is probably due to the special attention accorded to only *Aspergillus* mycotoxins (especially aflatoxins) in the region, thereby neglecting other toxins. Notably, there is an absence of regulations governing the control of *Fusarium* mycotoxins in SSA, thus subjecting the region to strictly depend on maximum levels in regulations and guidelines of the European Union (EU) and the Codex Alimentarius Commission (CAC) on *Fusarium* mycotoxins without putting into considerations the feeding habits, food security status, occurrence levels in the region, and the genetic/hereditary dispositions (genetic and environmental interactions) of the people that make up the region. The absence of regulation is thus attributed to the lack of sufficient scientific data (occurrence, exposure, and toxicological) and the socio-economic factors, such as public ignorance, hunger (as well as hidden hunger), as well as political and economic instability. It is noteworthy to mention that only a few of the countries in SSA have food control administrative systems that are functional. In most cases, the weak regulatory bodies are led (most often by political appointees) most times by personnel and stakeholders with minimal background knowledge about food toxins. The organization of academic conferences and workshops have not yielded many anticipated results. A good starting point will be the enhancement of knowledge and awareness. The availability of resource materials on *Fusarium* mycotoxins in public domain, setting up promotions, and the establishment of integrated driven interventions by government and stakeholders will definitely help to control these toxins. We propose a shift in action through the establishment of country or regional hub reference testing laboratories. This will go a long way in harmonizing efforts within countries while promoting a free flow of food products. Although proactive legal procedures on *Fusarium* mycotoxin control will certainly increase the burden of hunger with far reaching consequences, setting up *Fusarium* mycotoxins regulations in SSA would be a guiding pillar, principle, and requirement for food safety, and a mechanism to strengthen food control systems in the region.

This review focuses on the occurrence of *Fusarium* mycotoxins and their modified forms in SSA. In addition, the authors provide an overview of food processing control strategies regarding *Fusarium* mycotoxins, as well as future perspectives.

## 2. Occurrence of *Fusarium* Mycotoxins in Sub-Saharan Africa

The occurrence of *Fusarium* mycotoxins in agricultural products and its processed foods is of great concern because of its toxic effects in humans and animals. Global occurrence data on *Fusarium* mycotoxins have been reviewed [42,43,44], especially on the major mycotoxins (fumonisins, zearalenone, and trichothecenes) and their possible health effects [45,46]. Conversely, it is of concern that, of all the *Fusarium* mycotoxins existing, fumonisins are the only most studied in SSA, thus neglecting others and placing SSA as the least studied with respect to *Fusarium* mycotoxins research, irrespective of climatic change, food insecurity, poor prevention, and control strategies, and mycotoxin-poisoning problems ravaging in this region. Additionally, it is worth mentioning that most of the studies on *Fusarium* mycotoxins reported for the region were carried out in laboratories in the developed countries, thus buttressing a lack of infrastructural facilities required to conduct such studies in SSA. Other issues include the insufficient or lack of adequately trained personnel, as well as limited research investments in terms of funding for SSA research centers and academic institutions. As much as there is a need to reduce hunger within SSA (most especially in the resource poor communities), issues of food safety as regards *Fusarium* mycotoxin occurrence in foods and food products should be considered paramount.

### 2.1. Fumonisins

Fumonisins (FBs) were first described in South Africa by Bezuidenhout et al. [47]. About 28 FB analogs have been characterized and are classified into four main groups (A, B, C, and P series), with those belonging to the B series (FB_1_, FB_2_, and FB_3_) being the most abundant and of toxicological importance. Each FB in the B-series has a linear 20-carbon backbone with methyl, hydroxyl, and tricarboxylic acid moieties at various positions along the backbone. FB compounds are produced by a large array of *Fusarium* species, such as *F. verticillioides* and *F. proliferatum*. However, the production of FB by *Alternaria alternataf*. sp. *lycopersici* and *Aspergillus niger* has also been reported [48,49]. Ingestion of FB has been associated with several human and animal ailments worldwide because of their hepatotoxicity, nephrotoxicity, neurotoxicity, immune stimulation, and immune suppression, causing several developmental abnormalities, and liver and kidney malfunctions [50,51,52,53]. Human epidemiological studies have revealed a possible link of the consumption of FB-contaminated maize (corn) with esophageal cancer in South Africa, China, Northeastern Italy, and the southeast of the United States [50,54,55,56]. Cases of human abdominal pains and diarrhea were reported in India, resulting from consumption of moldy maize or sorghum containing high levels of FB [57]. Simultaneously, there is an assumption of a possible increase in the risk of neural tube defects because of the human maternal exposure to FB during the early stages of pregnancy [58]. Recently, the International Agency for Research on Cancer (IARC) has reported the possible association between fumonisin and stunting in children [59]. Cases of animal diseases as a result of FB have also been reported [51,60,61,62,63,64,65].

The occurrence of FBs has been reported in several cereals, legume crops, spices, and food products all over the world. Maize and its products remain the most contaminated because of the susceptibility of the maize crop to FB-producing fungi. In SSA, maize serves as a major cereal consumed on a daily basis by most of the population [38]. It is estimated that the average daily consumption rate of maize per adult is as high as 500 g. Occurrence data in SSA reveal high incidences and high levels of FB contamination of staple foods, especially maize (Table 1), which suggest that humans and animals in this region may be highly exposed to toxic effects unleashed by FB. In spite of the high occurrences and high levels of FB contamination and the fact that FB was first identified and characterized in South Africa [47], there remains a huge gap in research, leading to inadequate occurrence and toxicology data, and a lack of regulatory guidelines governing the control of this mycotoxin in SSA. Of the 49 countries in SSA, only a few have data on the occurrence of FB contamination in crops and food products (Table 1). However, because of the limited nature of the occurrence data reported so far, not a single country has established FB regulatory limits. SSA still depends on the recommended maximum levels set by the EU and the US Food and Drug Administration (FDA).

### 2.2. Trichothecenes

Trichothecenes (THs) are a large group of structurally related sesquiterpenoid mycotoxins produced by a wide range of *Fusarium* spp., although other mold genera such as *Trichoderma, Trichothecium*, *Stachybotrys*, *Verticimonosporium*, *Cephalosporium*, *Myrothecium*, and *Cylindrocarpon* can also synthesize them [102]. THs have a tetracyclic 12,13-epoxytrichothecene skeleton in common and are divided into four categories based on their chemical properties, which include type A, B, C, and D. Approximately 180 THs exist, but the ones of economic concern include those of type A (T-2 toxin (T-2), HT-2 toxin (HT-2), and diacetoxyscirpenol (DAS)) and type B (deoxynivalenol (DON), nivalenol (NIV), and fusarenon X (FX)) because of their frequent occurrence in food commodities and their toxic effects.

Ingestion of TH-contaminated food products have been associated with several human and animal diseases probably because of an epoxide at the C_12,13_ positions, which exhibits toxicological activity [103]. THs show varying degrees of cytotoxic potency based on the type, the dose, and the duration of exposure. They have been revealed as inhibitors of eukaryotic protein synthesis as well as DNA, RNA synthesis, and they affect cell division and inhibit mitochondrial function [5,104]. Prelusky et al. [105] and Rotter et al. [106] reported type A THs to be more acutely toxic, while those belonging to type B are implicated in more chronic toxicoses. Of all the THs, clinical data from animal studies suggest that T-2 and DAS are more potent [5]. In addition to inhibitors of eukaryotic protein synthesis, T-2 and HT-2 induce hematotoxicity, myelotoxicity, growth retardation, and necrotic lesion [107]. At low doses, DON exhibits toxicity often characterized in animals by feed refusal, thus decreasing growth rate. In higher exposure rate, it expresses immunosuppressant and immunostimulation properties. Epidemiological studies suggest the possibility of DON causing emetic effects in humans [108]. In addition, the study of Razafimanjato et al. [109] revealed the potential of DON decreasing the viability of glial cells responsible for maintaining brain homeostasis, thus causing modifications of brain homeostasis and possibly participating in the etiology of neurological diseases in which alteration of glial cells are involved. Similarly, NIV has been shown to exert clinical effects such as hematotoxicity and immunotoxicity in mammals. Possible symptoms of TH toxicity include vomiting, headache, dizziness, bleeding, nausea, fever, abdominal distress, dyspnea, and weight loss abortion, and may lead to death, although the symptoms may vary with animal species. An association of THs (T-2) with alimentary toxic aleukia in humans as a result of consumption of grains contaminated with *F. sporotrichioides* was reported in the Orenburg region in Russia and led to the death of thousands of people. A similar outbreak of a disease called akakabi-byo in Japan, as a result of consumption of *F. graminearum*-contaminated grains, was also reported by Marasas et al. [110]. Other outbreaks of acute poisoning in humans which exhibited symptoms such as vomiting, nausea, diarrhea, abdominal pain, dizziness, and headache as a result of consumption of *Fusarium*-contaminated grains have also been reported [111].

THs are commonly found in agricultural products, especially cereal crops such as wheat, maize, barley, oats, rye, rice, and other cereal-based foods, worldwide. Natural occurrence of DON in cereals is prevalent, and surveys from South America, Canada, China, and many countries of Europe have shown frequent occurrence, as well as high levels in cereal crops. In Europe, type B THs seem to be the most dominant [3], and this has expedited the establishment of regulatory limits for these toxins in various foodstuffs in order to avoid outbreaks of toxicoses [112]. Contrary to the state of research on THs in Europe and other parts of the world, SSA has paid little attention to TH research. Available published data (Table 2) suggest evidence of high occurrence of THs in SSA, and high concentrations up to 3842 μg/kg of DON in maize were reported as well [40]. Furthermore, the ability of THs to co-occur in food commodities as observed by some authors (Table 2) raises important issues regarding synergistic and/or additive effects in humans and animals [113]. However, the TH occurrence data in SSA is grossly inadequate, and this explains why there are no TH regulations despite the fact there is evidence of occurrence of these mycotoxins.

### 2.3. Zearalenone

Zearalenone (ZEN) is a secondary metabolite produced by a variety of *Fusarium* fungi species including *F. graminearum*, *F. culmorum*, *F. verticillioides*, *F. cerealis*, *F. equiseti*, *F. crookwellense*, and *F. semitectum*. These fungi contaminate crops in the field and thus produce ZEN prior to harvest. However, production of ZEN during storage have equally been reported by Kuiper-Goodman et al. [117], who observed high-level production of ZEN in maize-based feed as a result of improper storage. ZEN is commonly found in cereal crops, though its occurrence in other food products such as soybean products, dried fruit and vegetables, and cheese snacks have also been reported [118,119,120]. ZEN often co-occurs with other mycotoxins including DON, 15-acetyldeoxynivalenol (15-ADON), 3-acetyldeoxynivalenol (3-ADON), NIV, and FX because of the ability of the producing fungi to synthesize more than one mycotoxin which often results to synergistic and/or additive effects on the host organism [6].

ZEN is a non-steroidal estrogenic mycotoxin affecting both animals and humans. It has high binding affinities for the intracellular estrogen receptor and can enhance the proliferation of estrogen-responsive tumor cells [121]. Studies have reported the ability of ZEN to stimulate the growth of estrogen-responsive positive cells, increase uterine weight, modulate the estrous cycle and compete with estradiol for estrogen-responsive binding [122]. Its occurrence in foods and feeds has been linked to mammary tumorigenesis and hyperestrogenism, especially in pigs, resulting in adverse effects on the reproductive performance of breeding animals [123]. In addition, ZEN has been alleged to cause human cervical cancer and premature initial breast development [124], and an epidemic of precocious pubertal changes in young children in Puerto Rico between 1978 and 1981 [125]. Other authors also reported a possible link between ZEN and the incidence of esophageal cancer in certain parts of the world in combination with other mycotoxins such as fumonisins and trichothecenes [126,127]. Ingestion of ZEN has exhibited symptoms such as enlargement of mammary glands, vaginal and rectal prolapses, vaginal swelling (vulvovaginitis), testicular atrophy, infertility, prolonged estrus and reduced sexual drive, stillbirths, abortion, and reduced litter size [105,128]. Despite the wealth of information on the toxic effects of ZEN on the health of both humans and animals, and the evidence of high occurrence and levels of ZEN in food and food products exceeding the maximum limit set by the EU (Table 3), there is still a knowledge gap as regards the occurrence of ZEN in SSA.

### 2.4. Emerging and Modified Fusarium Mycotoxins

Studies on *Fusarium* mycotoxins have primarily focused on the occurrence and toxicological effects of FB, TH, and ZEN on humans and animals as well as prevention and detoxification strategies of these mycotoxins in food chains. However, in recent years, mycotoxin research has broadened upon other *Fusarium* mycotoxins such as fusaproliferin (FUS), beauvericin (BEA), enniatins (ENN), and moniliformin (MON) often called emerging *Fusarium* mycotoxins. Evidence of their occurrence in different food products has been reported [13,132,133,134,135,136,137,138], thus posing a severe challenge in some parts of the world. Little or no appreciable study has been carried out on the occurrence of these mycotoxins in food and food products in SSA (Table 4). Neglecting these *Fusarium* mycotoxins increases the risk of exposure of humans and animals to mycotoxin toxicity because of the possible high incidence and concentration in cereals and cereal-based products, which serve as staple foods in the region. BEA and ENN have shown cytotoxic and apoptotic effect on several humans cell lines and animal species [139,140]. They also act as specific inhibitors to the cholesterol acyltransferase [141,142]. In addition, ENN has been found to have a synergistic, additive, and antagonistic toxic effects on Caco-2 cells because of the possible co-occurrence of ENN analogues [143]. MON is a potent inhibitor of the pyruvate dehydrogenase complex, inducing cardiotoxicity, immunosuppression, muscular weakness, and intestinal problems [144,145,146]. On the other hand, FUS has exhibited teratogenic and pathogenic effects on human B-lymphocyte cells [147].

Apart from the emerging *Fusarium* mycotoxins, a recent concern is the occurrence of modified mycotoxins. These toxins are often not detectable by basic routine analytical methods, thus leading to an underestimation of the mycotoxin concentration. Modified mycotoxins may be matrix-associated or generated as a result of the modification of the chemical structure of free mycotoxins by plant, animal or fungus metabolism, or during food processing. However, some free mycotoxins may also be classified as modified mycotoxins, especially the acetylated DON (3- and 15-acetyldeoxynivalenol) [17]. It is noteworthy to highlight that, in an attempt to detoxify DON, plants genetic transformation by implementing a 3-O-acetyltransferase allows for the acetylation of DON to 3-acetyldeoxynivalenol (3-ADON), a trait which plants do not possess naturally, thereby classifying 3-ADON under biologically modified mycotoxin [148,149]. Several studies have revealed the occurrence of modified forms of ZEN, TH, and FB mycotoxins in cereals, cereal-based food, and feed products [19,21,113,150,151,152]. However, there seems to be little or no available data on modified mycotoxins in SSA (Table 4). Though toxicological data are still limited, the occurrence of modified mycotoxins is extrapolated to add substantially to the overall mycotoxins levels and toxicity. The increase in toxic health effects by modified mycotoxins may be either direct or indirect via hydrolysis, or released from the matrix during digestion into the free compounds [153]. Comparative cytotoxic effects of DON and its acetylated derivatives on a non-transformed intestinal epithelial cell line using 3-(4,5-dimethylthiazol-2-yl)-2,5-diphenyltetrazolium bromide (MTT) revealed that 3-ADON exerted less toxic effects (EC_80_ value of 125 µM) when compared to the free toxin (EC_80_ value of 16.5 µM) while the reverse was the case with 15-ADON (EC_80_ value of 10.5 µM) [154]. Their findings were in agreement with the previous studies of Pinton et al. [155] and Kadota et al. [156], who compared the toxicity of DON, 3-ADON, and 15-ADON on porcine intestinal epithelial cells and human intestinal Caco-2 cell, respectively. Pinton et al. [155] reported a reduction in cell proliferation by DON and its acetylated derivatives in the ranking order of 3-ADON (13%) < DON (60%) < 15-ADON (69%), while Kadota et al. [156] observed the same trend on the interleukin-8 production in Caco-2 cell. Further ex vivo (porcine intestinal explants) and in vivo (jejunum from piglets) analysis showed that 15-ADON exerted more toxicity than DON and 3-ADON [155]. A much earlier study by Forsell et al. [157] buttressed this view when mice were exposed to acute oral toxicity of DON and 15-ADON. DON and its metabolites are able to increase the permeability of the intestinal epithelial layer by decreasing the expression of tight junction proteins [152]. This can be worsened by a reduction in cell proliferation, thus increasing susceptibility to pathogens. While the consistency of results suggests a trend, these protocols need to be replicated in different animal models while minimizing variations within experimental units. The co-contamination of DON with other mycotoxins and metabolites of DON, and their potential synergistic and additive effects remains a knowledge gap. Eriksen et al. [158] compared DON, its acetylated and deepoxy metabolites on Swiss mouse 3T3 fibroblasts. Their findings showed a similar lower toxic effect by 3-ADON, whereas DON and 15-ADON had equal effects. De-epoxy deoxynivalenol (DOM) was 50 times less toxic than DON [158]. The reduced toxic effect of DOM is attributed to the de-epoxidation of the 12,13-epoxy ring in the structure of TH which is the essential functional group alleged to cause toxicity [159]. Regarding glucosylated form of DON, Pierron et al. [160] studied the possible toxic effect of deoxynivalenol-3-glucoside (DON-3G) in comparison with DON on the intestine using the human intestinal Caco-2 cell line and porcine jejunal explants. Their investigation revealed the inability of DON-3G to bind to the ribosome, thus decreasing its intestinal toxicity when compared to DON. This is in line with the in vitro cytotoxicity study of DON-3G on porcine intestinal epithelial cells ranking DON-3G as the least toxic compared to DON and its acetylated forms [161]. In addition, an in vivo study by Broekaert et al. [162] demonstrated that DON-3G has a low absolute oral bioavailability in broiler chickens compared to DON. DON-3G is not hydrolyzed to DON in broiler chicken similar to the trend reported in different in vitro studies [163,164]. In contrast with the study on broiler chicken, Broekaert et al. [162] observed a different trend when pigs were orally administered with DON-3G demonstrating a complete hydrolysis of DON-3G to DON although the absorbed fraction was approximately 5 times lower than after oral administration of DON [162]. While in vitro studies of DON-3G suggest less toxic effects, the latter study on pig proves that the toxicological significance of DON-3G should not be neglected especially across different animal species. Apart from the ability of modified metabolites of DON to exert direct toxic effect on animal or human host, a major concern is their hydrolyses into their free forms after ingestion. Studies have reported the possible potentials of these metabolites being hydrolyzed to their free forms [15,16]. In order to understand the transformation of 3-ADON and 15-ADON to DON, Ajandouz et al. [16] studied the deacetylation activity of 3-ADON and 15-ADON by enzymes, bacteria, cells, and tissues present in humans. Interestingly, they observed that 3-ADON was more prone to deacetylation than 15-ADON, while small intestine and liver are the major sites of deacetylation of 3-ADON and 15-ADON in humans. The toxicity of 4-acetyl NIV (FUS X) on Swiss mouse 3T3 fibroblasts showed that 4-acetyl NIV exhibited 1.7 times more toxic effects than NIV [158]. This trend was also observed in previous studies by Visconti et al. [165], and Eriksen and Alexander [166]. These findings were in line with the study of de-epoxy T-2 using rat skin irritation assay. Their result showed that de-epoxy T-2 exhibited 400 times less toxic effect than the corresponding T-2 [167]. Similarly, the cytotoxicity effects of ZEN and its major metabolites alpha-zearalenol (α-ZEL) and beta-zearalenol (β-ZEL) on cultured human Caco-2 cells revealed variable toxic effects of ZEN and its metabolites with observation showing that the toxic effects seem to be relieved by the metabolism of ZEN into α-ZEL and β-ZEL [168]. Othmen et al. [169] reported that α-ZEL and β-ZEL inhibited cell viability, protein and DNA syntheses, and induced oxidative damage, and over-expression of stress proteins. However, α-ZEL and β-ZEL exhibited lesser toxicity than ZEN, with β-ZEL being the more active of the two metabolites. A reverse toxicity trend was observed in the estrogenic potential of these compounds [170], with α-ZEL being ranked as the most toxic, followed by ZEN, and then β-ZEL. This trend of toxic effect was shown by Ayed et al. [171]. Zearalenone-14-sulfate (ZEN-14S) and zearalenone-14-glucoside (ZEN-14G) exhibited low estrogenic potential which is attributed to their inability to bind to the estrogen receptor [172,173]. Apart from the low estrogenic potential of ZEN-14G, an in vitro study also showed a lower toxicity of ZEN-14G with respect to its free form (ZEN). Dellafiora et al. [174] studied the hydrolysis of ZEN-14G to its free form (ZEN) in the bovine blood and blood components including plasma, serum, and serum albumin. Their study revealed the reduction in ZEN-14G in all the treatments, thus leading to the release of ZEN with a significant amount of zearalenol isomers (α-ZEL and β-ZEL) in whole blood.

While there are evidences of the occurrence of modified mycotoxins in food and feed products, it is presently impossible to establish regulations that protect consumers because of a lack of exposure and toxicological data. Studies on this subject have been fragmented and hence unable to make quantum leaps in filling the voids of unanswered questions. This necessitates an urgent need for more research on the occurrence and the potential health effect of modified mycotoxins, as well as understanding the behavior of modified mycotoxins during food processing. In our view, standardization of experimental protocols, and clinical testing across laboratory and regions, is critical and timely. More research efforts should be geared toward the development of reference standards for modified mycotoxins. This will offer a platform for easy detection and quantification of modified mycotoxins in food and food products across the globe.

## 3. Mitigation Strategies of *Fusarium* Mycotoxins during Processing

Over the years, the scientific community has proposed good agricultural practices (GAP), followed by implementation of good manufacturing practices (GMP), and hazard analysis and critical control points (HACCP) during food processing as a strategic measure in addressing the problems posed by fungi and mycotoxins in the food system. Food processing may be physical (cleaning and milling processes, physical adsorption, and thermal processes), chemical (use of ammonia, calcium hydroxide, and sulfur containing compounds) or biological (malting, brewing, and fermentation). The degree of reduction in mycotoxin concentrations in food crops and feeds by processing is dependent on the matrix type, the mycotoxin, as well as the processing method, and different conditions employed. Besides studying the effect of processing on mycotoxins, it is important to be aware of the possibility of free mycotoxins co-occurring with their modified forms, or the free mycotoxins being modified and fragmented into other forms during food processing, which may not be easily detected by routine methods. A lack of awareness of these mitigation processes have prevented SSA from progressively reducing mycotoxins in foods and feeds. Creating awareness on the effect of implementation of GAP, GMP, and HACCP in the control of toxic metabolites in the food system will be ideal to some extent in reducing the risk of mycotoxins exposure in both the rural and urban communities in SSA.

### 3.1. Cleaning and Milling

Cleaning and sorting are considered to be the first step of physical decontamination. These techniques are regarded as superior methods because they pose no risk of producing degradable products which subsequently may be toxic [176]. These methods are dated back as old as the beginning of mankind. Several studies have reported the efficiency of physical decontamination methods such as sorting, washing, dehulling, and removal of visible moldy and floating kernels in the reduction of different types of mycotoxins in foods irrespective of the grain type [91,177,178,179,180,181,182,183]. Reduction between 26% and 69% of total FB in maize was observed by Sydenham et al. [179] as a result of cleaning, prior to further processing. A 32% reduction in FB levels in maize in an industrial mill was also reported by Scudamore and Patel [184]. The same trend was observed by Van der Westhuizen et al. [182], who recorded a reduction range of 27%–93% of FB after sorting contaminated maize. Furthermore, Pascale et al. [183] and Scudamore and Patel [185] observed a reduction of T-2 (62%) and HT-2 (53%), and DON (50%) in wheat grains after cleaning. The reduction recorded by these authors may be ascribed to the fact that mycotoxins are often concentrated in dust and broken kernels because of their susceptibility to fungal infection and subsequent mycotoxin production. Thus, the percentage of mycotoxin reduction by cleaning and sorting of grains is determined by the physical condition of the grains, as well as the type and effectiveness of the cleaning method. In addition, milling plays a potential role in the reduction of *Fusarium* mycotoxins in grains. However, the problem often encountered is the differential toxicity of the fractions resulting from grain separation. Lee et al. [186], Dexter et al. [187], and Lancova et al. [188] registered the reduction of DON during milling of wheat. This was in agreement with the study of Tibola et al. [189], who reported a higher deposition of *Fusarium* mycotoxins in wheat bran after milling. A similar trend was observed with respect to emerging *Fusarium* mycotoxins. A reduction of 71% and 79% of ENN B and ENN B_1_ in wheat flour, respectively, was recorded by Vaclavikova et al. [190] as a result of milling, with the highest concentrations of ENN B and ENN B_1_ being detected in the bran and shorts. Moreover, similar results were also reported regarding distribution of modified mycotoxins in cereals after milling [189,191,192]. The study on the fractionation of DON and DON-3G in milling fractions showed a similar trend with white flours containing approximately 60% of the content in unprocessed wheat grains [191]. The reduction reported by these authors is attributed to the fact that, during dry milling, the highest amounts of mycotoxins are concentrated in the fractions of the commodity (bran) that are less likely to be used for food production, though these higher contaminated fractions mostly end up as animal feed. Furthermore, wet milling of maize has shown to result in the reduction of mycotoxins. Mycotoxins may dissolve into the steep water or be distributed among the by-products while the starch remains relatively free from mycotoxins [193,194,195].

### 3.2. Thermal Treatment

Several other methods such as thermal treatment used in food processing have been studied to understand its effects on mycotoxins. Mycotoxins are generally heat stable and as such are not easily destroyed during most normal cooking processes [196,197]. However, at very high temperatures, reduction has been reported to occur although this may be as a result of reactions resulting in the formation of products with altered chemical structures. Ryu et al. [198] proved the effectiveness of thermal treatment (extrusion cooking) on the reduction between 66% and 83% of ZEN at temperature ranging from 120 °C to 160 °C. Scott and Lawrence [199] also reported 60%–100% reduction of FB when heating dry and moist corn meal at 190 °C (60 min) and 220 °C (25 min) respectively. In addition, Shephard et al. [200], in their study using a traditional South African method for production of maize porridge, observed about 23% reduction in FB concentration. Notwithstanding the FB reduction reported during thermal processing, it is important to state the frequent occurrence of bound FB in thermally treated foods because of the binding of FB with matrix constituents through covalent interaction at high temperatures via a Maillard-type reaction [201]. This is evidenced in the studies available on the effect of thermal treatment on FB, which indicated that the largest reduction of FB occurs at a temperature of 160 °C or more in the presence of glucose [202]. The main products were *N*-carboxymethyl FB and *N*-deoxyfructosyl FB although upon alkali treatment, a hydrolyzed form may be formed by the cleavage of both carballylic moieties [202]. These bound FBs are not detectable by the basic routine analytical methods, which may thus explain the reduction reported.

In the case of TH such as DON, there have been lots of contradicting reports by different authors on the effect of thermal processing. While Bergamini et al. [203], Kostelanska et al. [191], Numanoglu et al. [204], and Vidal et al. [205] reported a reduction in DON content in bread; Lancova et al. [188] and Scudamore et al. [206] recorded no effect in DON concentration by thermal processes. This conflicting disparity may be attributed to varying baking temperatures, baking procedures, and ingredients used. Furthermore, the analytical methods used and experimental conditions may have contributed to the variation in the trends observed by these scientists. Interestingly, De Angelis et al. [207] documented an approximately 18% higher level of DON in bread when compared to the original flour, which is in line with the study of Young et al. [208]. The increase may be explained by the release of DON from their modified forms of DON-3G. This phenomenon corresponded with the significant drop in DON-3G levels in bread, and may be due to the activities of yeast during fermentation. In contrast, Vidal et al. [205] reported an increase in DON-3G during baking. Moreover, the same authors investigated the effect of bread baking on T-2, HT-2, and their glucoside conjugates and observed a reduction in the concentration of T-2 (range: 63%–74%) in bread as compared with the original flour, while HT-2 levels appeared to be less affected [207]. A reduction of T-2 may be ascribed to the partial conversion of T-2 to HT-2 during yeast fermentation operated by the carboxylesterase naturally present in cereal-based products and/or partial degradation of T-2 due to thermal treatment. In the case of T-2 glucoside (T-2G) and HT-2 glucoside (HT-2G), the same trend was recorded in HT-2G, while a reverse behavior was found for T-2G. These results agreed with the report of Humpf and Voss [202] on the possible formation of unknown biologically active compounds or the reversible binding of the toxin to sugars or proteins in the food/feed matrix during heat treatment.

### 3.3. Fermentation

Another universal biological food processing method is fermentation. In SSA, fermentation is one of the most technologically appropriate methods for food processing because of its affordability and suitability for the production of staple foods in rural and urban regions. Although fermentation offers many advantages such as food preservation, enhanced sensory qualities, increased nutritional value and variety of food type, reduced anti-nutritional compounds, improved functional properties, and food safety, the living cells and enzymes used during this process may lead to the liberation or transformation of mycotoxins into modified mycotoxins. Furthermore, *Fusarium* fungi when present during fermentation, are still capable of growing and synthesizing mycotoxins. Information on the effect of fermentation on *Fusarium* mycotoxins, especially using the African traditional fermentation methods, is limited. Diverse results have been reported by different authors on the effect of fermentation on mycotoxins (especially DON) during bread making. While some studies recorded a mean reduction of DON in fermented dough [209,210,211], others reported stability [212] and an increase in DON concentration during fermentation [203,205,208], although the increase observed by Vidal et al. [205] was a combined effect of kneading, fermentation, and proofing. These conflicting reports could be a result of several factors such as differences in technology, process temperature, and initial concentration of the mycotoxins. Interestingly, the possible explanation of the increase in DON concentration as reported by the latter authors may be a result of the enzymatic release of bound forms of DON occurring in the raw materials. Kostelanska et al. [191] reported an increase of up to 145% in DON-3G in the fermented dough when a bakery improver’s enzymes (16% of protease, 39% of xylanase) were added. A similar report on the increase in DON (3.5%) after fermentation was observed in wheat germ-enriched bread [213].

A study on the effect of fermentation on mycotoxins during local processing of Nepalese traditional beer using experimentally contaminated maize showed stability of FB_1_ throughout the fermentation process, while a 50% reduction in DON was recorded [214]. In contrast, Bothast et al. [215] observed a low reduction of FB_1_ during the fermentation of naturally contaminated maize for ethanol production. Ezekiel et al. [216] reported high-percentage (99%, 100%, 98%, 98%, and 76%) reductions of DON, FB, fusaproliferin (FP), MON, and ZEN in fermented Nigerian cereal-based beverages (kunu-zaki and pito), respectively. However, their result showed a much higher reduction in maize-based beverage (kunu-zaki), when compared to sorghum-based beverage (pito) because the raw maize was more contaminated than the raw sorghum. This proves that the degree of the reduction of mycotoxins in foods or feeds is dependent on the initial mycotoxin concentrations. In a recent study on the effect of malting process on *Fusarium* mycotoxins, the authors observed a similar behavior of DON, 3-ADON, and 15-ADON throughout the malting process, while steeping reduced the concentration of DON, 3-ADON, and 15-ADON between 15% and 49% of the initial level independent of the cultivar and inoculation type [217,218]. In contrast, Kostelanska et al. [219] and Habler et al. [218] observed an opposite effect on DON-3G after germination, apparently because of the induction of glycosylation of DON by DON-glycosyl-transferase enzyme during germination [217]. In view of the conflicting data reported by different studies on the effect of processing methods on mycotoxins, there is a need for further studies to harmonize and fully understand the behavior of mycotoxins during food processing.

## 4. Future Perspectives for Sub-Saharan Africa

The economic and health hazards of mycotoxin contamination in crops and food products present a huge challenge, especially in SSA, where there is limited data to ascertain the degree of harm caused by these toxins. Tackling this problem needs a multi-factorial approach. A workable strategy would be the systematic development of centers of research expertise, and building research capacities aimed at establishing a database on health-related risks caused by mycotoxins. Growing the interest of the African scientific community towards increasing the research output in the region is imperative. To this end, building a human resource capacity on mycotoxicology is a good starting point. National and regional hubs of excellence can be used as a platform. This will ensure a coordinated response approach, while postgraduate training using state-of-the-art infrastructure will ensure sustainability. The present analytical methods in SSA may not provide accurate measurements of total contamination in food crops and products, and may underestimate the actual levels. The lack of data precision and reproducibility is creating a significant bottleneck to progress. Recent advances in chromatographic techniques such as high-performance liquid chromatography (HPLC), liquid chromatography-mass spectrometry (LC-MS), and high-resolution mass spectrometry (HR-MS) provide more choices and options to improve analytical performance. However, this is not the case in SSA as these instruments are rarely available, and when available, there is difficulty in maintaining and servicing due to epileptic power voltage, unskilled manpower for instrumentation maintenance, and absence of technical outlets of the manufacturing companies in the region. The establishment of technical offices within the national and regional hubs as proposed above will cushion these effects. In addition, developing the technical expertise of African nationals with respect to maintenance and management of these sensitive analytical instruments will be more pro-active, and in the long run more beneficial to SSA. In the midst of these challenges, the development of simple, precise, and low-cost diagnostic tests such as ELISA and lateral flow immunoassay (LFIA) can foster better mycotoxin monitoring in SSA. Governments within the region need to ensure a stability in policy, economics, and political environment to guarantee investment. An accelerated human capacity and infrastructural growth in mycotoxins research is proposed. The systemic institutional weakness of existing food regulatory agencies in SSA can be circumvented through the stakeholders’ advocacy and regional partnerships. The establishment of a mycotoxin community of practice as well as the strengthening of mycotoxicology scientific meetings represent a good starting point.

Another great challenge for the next decade is to mitigate the effect of climate change on crop production with a focus on sustaining crop and animal production levels with reduced contamination. A multi-pronged approach of using a combined expertise will be critical in sustaining a healthy food intake most especially in SSA. Management strategies need to put into perspective the influence of input control measures of mycotoxigenic pathogens, the influence of environmental phenomena, the prevalence of non-symptomatic crops with toxin contamination, and the prevalence of quantitative resistance crops to both pathogen infections and toxin production. Furthermore, concerted efforts are required by farmers, post-harvest food specialists, breeders, agronomists, and technologists toward precise and strategic management systems with respect to the diverse staple food systems in the region.

The technical institutional and policy intervention measures are non-existent in most countries within SSA. Establishment of these frameworks with legal backup will help in tackling problems that might arise in the context of screening the commodity value chain. We think there is no systemic surveillance of *Fusarium* mycotoxin diversity in toxigenic fungi in SSA. Such studies have been highly fragmentary. A regular surveillance survey in this regard will add value to already known knowledge, while bringing to the fore a better understanding of the depth of problems inherent in SSA. An understanding of the evolutionary dynamics of these toxins is mostly needed. Breeding for field crop quantitative resistance is yet another option. Most often, field crop breeders are biased towards yield and disease resistance. An integrated team of postharvest specialists and mycotoxicologists should be part of the screening or phenotyping process of the breeder. Varietal releases should incorporate some sort of quantitative resistance to toxigenic fungi. Looking beyond the conventional breeding effort, genetic engineering can be exploited where specific genes of interest can be integrated to mitigate or prevent toxigenic progression of most fungi. Biological control measures using competitive exclusion principles in the various cropping systems can be exploited. This proved efficient in the control of pathogens [220] as exhibited by the use of atoxigenic strains of *Aspergillus flavus* to control aflatoxin producing *A. flavus* [221].

## 5. Conclusions

Although there is wealth of information on *Aspergillus* mycotoxins, especially the aflatoxins in SSA, the reverse remain the case with the *Fusarium* mycotoxins as revealed in this review. The knowledge gap as regards *Fusarium* mycotoxin research in SSA is of concern because of the frequent occurrence and co-occurrence of these toxins in staple food and food products. Few studies conducted on the occurrence of the major *Fusarium* mycotoxins (FB, TH, and ZEN) in food and food products in SSA revealed possible high levels of these toxins, in most cases exceeding the maximum limit set by regulatory agencies. A recent concern is the occurrence of emerging and modified *Fusarium* mycotoxins in food and feed commodities. Although the metabolic fate of modified mycotoxins still remains a matter of scientific discourse, SSA must not be left behind. Existing reports on in vitro and in vivo metabolic studies of modified mycotoxins prove that these toxins may be hydrolyzed to the free toxins in the gastrointestinal tract, thereby indicating potential toxic relevance on the host species. As such, there is need for constant and continuous monitoring of the occurrence of *Fusarium* mycotoxins and their modified forms in food and feed commodities as some form of prevention as food quality in SSA improves.

## Figures and Tables

**Table 1:** Occurrence and contamination levels of fumonisins in food crops and products in sub-Saharan Africa since the year 2000.

Country	Commodity	Toxin Type	No of Sample	Sample Preparation	Technique	% Positive	Range (µg/kg)	Reference
Botswana	Sorghum malt	FB_1_	46	SPE (SAX)	HPLC	6.5	47–1316	[66]
Burkina Faso	Maize	FB_1_ + FB_2_	124	NA	HPLC	100	10–16,040	[67]
	Maize	FB_1_	26	NA	HPLC/ESI-MS/MS	81	22.5–1343	[68]
	Maize	FB_2_	26	NA	HPLC/ESI-MS/MS	69	11.3–589	[68]
	Feed	FB_3_	26	NA	HPLC/ESI-MS/MS	46	23.2–274	[68]
	Feed	FB_1_	4	NA	HPLC/ESI-MS/MS	75	578–3390	[68]
	Feed	FB_2_	4	NA	HPLC/ESI-MS/MS	75	186–1235	[68]
	Feed	FB_3_	4	NA	HPLC/ESI-MS/MS	75	70.0–362	[68]
	Others	FB_1_	30	NA	HPLC/ESI-MS/MS	4	73.8 (median)	[68]
	Others	FB_2_	30	NA	HPLC/ESI-MS/MS	4	28.2 (median)	[68]
Cameroon	Maize	FB_1_	40	SPE(SAX)	HPLC	65	37–24,225	[69]
	Maize	FB	165	SPE (amino)	UPLC-MS/MS	74	20–5412	[40]
	Maize	FB	18	NA	ELISA	88.9	nd–26,000	[70]
	Peanut	FB_1_	16	SPE (SAX)	HPLC	18.8	25–1498	[69]
	Bean	FB_1_	15	SPE (SAX)	HPLC	20	28–1351	[69]
	Soybeans	FB_1_	5	SPE (SAX)	HPLC	40	25–365	[69]
	Sorghum beer	FB_1_	120	SPE (C_18_)	ELISA	87.5	0–340 μg/L	[71]
	Maize	FB_1_	37	NA	LC-MS/MS	100	2–2313	[72]
	Groundnut	FB_1_	35	NA	LC-MS/MS	51	0.4–10	[72]
	Soybean	FB_1_	10	NA	LC-MS/MS	100	38–69	[72]
	Maize beer	FB_1_	14	NA	LC-MS/MS	100	15–741	[72]
	Groundnut soup	FB_1_	15	NA	LC-MS/MS	73	0.6–17	[72]
	*Kuru-Kuru*	FB_1_	6	NA	LC-MS/MS	100	2–4.3	[72]
	*Dagwa*	FB_1_	8	NA	LC-MS/MS	100	47–132	[72]
	Maize	FB_2_	37	NA	LC-MS/MS	97	7–572	[72]
	Groundnut	FB_2_	35	NA	LC-MS/MS	34	0.4–4	[72]
	Soybean	FB_2_	10	NA	LC-MS/MS	100	7–19	[72]
	Maize beer	FB_2_	14	NA	LC-MS/MS	100	0.6–127	[72]
	Groundnut soup	FB_2_	15	NA	LC-MS/MS	33	<LOQ–6	[72]
	*Dagwa*	FB_2_	8	NA	LC-MS/MS	100	15–37	[72]
	Maize	FB_3_	37	NA	LC-MS/MS	95	<LOQ–157	[72]
	Groundnut	FB_3_	35	NA	LC-MS/MS	43	<LOQ–5	[72]
	Soybean	FB_3_	10	NA	LC-MS/MS	100	2.3–14	[72]
	Maize beer	FB_3_	14	NA	LC-MS/MS	100	0.7–100	[72]
	Groundnut soup	FB_3_	15	NA	LC-MS/MS	7	1.88 (mean)	[72]
	*Kuru-Kuru*	FB_3_	6	NA	LC-MS/MS	33	3.4–4.2	[72]
	*Dagwa*	FB_3_	8	NA	LC-MS/MS	88	<LOQ–11	[72]
	Maize	FB_6_	37	NA	LC-MS/MS	27	1036–4368	[72]
	Maize beer	FB_6_	14	NA	LC-MS/MS	7	76.13 (mean)	[72]
	Groundnut soup	FB_6_	15	NA	LC-MS/MS	13	172–229	[72]
Côte d’Ivoire	Corn	FB_1_	10	NA	ELISA	100	300–1500	[73]
	Peanut	FB_1_	10	NA	ELISA	70	<300–6000	[73]
Democratic Republic of Congo	Maize	FB	40	SPE (SAX), IAC	TLC, HPLC	100	17.5–6258	[74]
	Bean	FB	30	SPE (SAX), IAC	TLC, HPLC	83.3	3.2–321	[74]
Ethiopia	Sorghum	FB	39	NA	ELISA	7.7	1370–2117	[75]
	Maize	FB	17	NA	ELISA	23.5	300–2400	[76]
	Sorghum	FB_1_	70	NA	HPLC/ESI-MS/MS	14.3	nd–30.1	[77]
	Sorghum	FB_2_	70	NA	HPLC/ESI-MS/MS	8.57	nd–8.4	[77]
	Sorghum	FB_3_	70	NA	HPLC/ESI-MS/MS	1.43	nd–2.5	[77]
	Millet	FB_1_	34	NA	HPLC/ESI-MS/MS	45.5	nd–49.2	[77]
	Millet	FB_2_	34	NA	HPLC/ESI-MS/MS	27.3	nd–16.1	[77]
	Millet	FB_3_	34	NA	HPLC/ESI-MS/MS	3.44	nd–6.3	[77]
Ghana	Maize	FB	15	SPE (SAX)	HPLC	100	70–52,670	[78]
	Maize	FB	75	SPE (SAX)	HPLC	90.7	11–2500	[79]
	*Kenkey*	FB	75	SPE (SAX)	HPLC	73.3	15–1000	[79]
Kenya	Maize beer	FB	61	NA	QuickTox Kit	9.8	280–4000	[80]
	Kenyan Lager Beers	FB_1_	75	SPE (C_18_)	ELISA	72	0–0.78 μg/L	[81]
Malawi	Maize	FB	90	NA	HPLC/ESI-MS/MS	90	nd–6475	[82]
	Maize beer	FB	9	SPE (C_18_), MultiSep	UPLC-MS/MS	100	1898 (mean)	[83]
Mozambique	Maize	FB_1_	13	NA	HPLC/ESI-MS/MS	92.3	159–7615	[68]
	Maize	FB_2_	13	NA	HPLC/ESI-MS/MS	92.3	27.7–3061	[68]
	Maize	FB_3_	13	NA	HPLC/ESI-MS/MS	85	26.6–777	[68]
	Feed	FB_1_	10	NA	HPLC/ESI-MS/MS	70	810–20,579	[68]
	Feed	FB_2_	10	NA	HPLC/ESI-MS/MS	80	13.5–7088	[68]
	Feed	FB_3_	10	NA	HPLC/ESI-MS/MS	70	94.3–2264	[68]
	Others	FB_1_	7	NA	HPLC/ESI-MS/MS	43	273–45,450	[68]
	Others	FB_2_	7	NA	HPLC/ESI-MS/MS	57	11.5–15,254	[68]
	Others	FB_3_	7	NA	HPLC/ESI-MS/MS	43	74.8–5115	[68]
Nigeria	Maize	FB_1_	70	NA	HPLC/ESI-MS/MS	92.9	1.8–10,447	[39]
	Maize	FB_2_	70	NA	HPLC/ESI-MS/MS	92.9	12.8–3455	[39]
	Maize	FB_3_	70	NA	HPLC/ESI-MS/MS	92.9	6.4–720	[39]
	Maize	FB_1_	182	NA	LC-MS/MS	73	10–760	[84]
	Maize snack	FB	8	NA	HPLC/ESI-MS/MS	100	4.8–339	[85]
	Groundnut-Maize snack	FB	2	NA	HPLC/ESI-MS/MS	100	12.5–130	[85]
	Maize	FB_1_	103	SPE (SAX)	HPLC	78.6	70–1780	[86]
	Maize	FB_2_	103	SPE (SAX)	HPLC	66	53–230	[86]
	Maize	FB_1_	108	NA	HPLC	50.9	65–1800	[87]
	Maize	∑FB	136	SPE (C_18_), MultiSep	LC-MS/MS	65	32–8508	[88]
	Sorghum	∑FB	110	SPE (C_18_), MultiSep	LC-MS/MS	8	45–180	[88]
	Millet	∑FB	87	SPE (C_18_), MultiSep	LC-MS/MS	14	74–22,064	[88]
	*Ogi*	∑FB	30	SPE (C_18_), MultiSep	LC-MS/MS	93	125–3557	[88]
	Rice	FB_1_	21	NA	HPLC	14.3	0.4–4.4	[89]
	Rice	FB_2_	21	NA	HPLC	4.8	132.5 (mean)	[89]
	Poultry feed	FB_1_	58	NA	LC/ESI–MS/MS	83	31–2733	[90]
	Poultry feed	FB_2_	58	NA	LC/ESI–MS/MS	81	51–1130	[90]
	Poultry feed	FB_3_	58	NA	LC/ESI–MS/MS	76	37–369	[90]
	Poultry feed	FB_4_	58	NA	LC/ESI–MS/MS	67	18–115	[90]
Republic of Benin	Maize	FB	36	IAC	fluorometer	100	600–2400	[91]
	Maize	FB	48	IAC	fluorometer	NA	nd–12,000	[92]
	Cassava flour	FB_1_	4	SPE (amino)	UPLC-MS/MS	100	4–24	[93]
	Maize	FB_1_	4	SPE (amino)	UPLC-MS/MS	100	51–836	[93]
	Maize	FB_2_	4	SPE (amino)	UPLC-MS/MS	100	5–221	[93]
	Maize	FB_3_	4	SPE (amino)	UPLC-MS/MS	100	<LOQ–375	[93]
	Maize	FB_1_	54	IAC	LC-MS/MS	100	56–14,990	[38]
	Maize	FB_2_	54	IAC	LC-MS/MS	100	38–6444	[38]
South Africa	Maize	FB	40	SPE (SAX)	HPLC	100	64–1035	[37]
	Maize	FB_1_	54	SPE (SAX)	HPLC	87	101–53,863	[94]
	Maize	FB_1_	96	SPE (SAX)	HPLC	38	100–22,200	[95]
	Maize porridge	FB_1_	47	SPE (SAX)	HPLC	74	0.2–20	[94]
	Compound feeds	FB	92	SPE (SAX)	HPLC	88	104–2999	[96]
	Cooked maize	FB_1_	28	SPE (SAX)	HPLC	29	100–400	[95]
Tanzania	Maize	FB_1_ + FB_2_	120	SPE (SAX)	HPLC	52	61–11,048	[97]
	Maize	FB_1_	60	NA	UHPLC/TOFMS	73.33	16–18,184	[98]
	Maize	FB_2_	60	NA	UHPLC/TOFMS	48.33	178–38,217	[98]
Zambia	Maize	FB	114	NA	ELISA	100	33,500–192,000	[99]
Zimbabwe	Maize	FB_1_	95	SPE (amino)	LC-MS/MS	95	nd–1106	[100]
	Maize	FB_2_	95	SPE (amino)	LC-MS/MS	31	nd–334	[100]
	Maize	FB_3_	95	SPE (amino)	LC-MS/MS	3	nd–67	[100]
	Maize	FB_1_	5	NA	HPLC	100	4000–8000	[101]
	Wheat	FB_1_	5	NA	HPLC	100	2500–6000	[101]
	Sorghum	FB_1_	5	NA	HPLC	100	200–1400	[101]
	*Rapoko*	FB_1_	5	NA	HPLC	100	300–2000	[101]
	Peanut	FB_1_	4	NA	HPLC	25	0–1000	[101]

Burkina Faso, others = 30 (sorghum—7, millet—3, rice—3, sesame—2, wheat—1, infant food formulations—3, mixed cuscus—3, cornflakes—2, cookies—2, and dried fruits–4); Mozambique, others = 7 (millet—2, soy—3, waste product from feed production—2); nd = not detected; NA = not applicable; LOQ = limit of quantification; FB = fumonisin; ∑FB = sum of FB_1_, B_2_, and B_3_; IAC = immunoaffinity column; SPE = solid phase extraction; SAX = strong anion exchange; HPLC/ESI-MS/MS = liquid chromatography/electrospray ionization tandem mass spectrometry; ELISA = enzyme-linked immunosorbent assay; UHPLC/TOFMS = ultra-high performance liquid chromatography/time-of-flight mass spectrometry; LC-MS/MS = liquid chromatography-tandem mass spectrometry; TLC = thin layer chromatography.

**Table 2:** Occurrence and contamination levels of trichothecenes in food crops and products in sub-Saharan Africa since the year 2000.

Country	Sample Type	Toxin Type	No of Samples	Sample Preparation	Technique	% Positive	Range (μg/kg)	Reference
Burkina Faso	Maize	DON	26	NA	HPLC/ESI-MS/MS	4	31.4 (median)	[68]
	Others	DON	30	NA	HPLC/ESI-MS/MS	33.33	22.3–250	[68]
	Others	NIV	30	NA	HPLC/ESI-MS/MS	3.33	40.2 (median)	[68]
Cameroon	Maize	DON	18	NA	ELISA	77.8	nd–1300	[70]
	Maize	DON	40	SPE (SAX)	HPLC	72.5	18–273	[69]
	Peanut	DON	16	SPE (SAX)	HPLC	75	17–270	[69]
	Bean	DON	15	SPE (SAX)	HPLC	46.7	13–35	[69]
	Soybeans	DON	5	SPE (SAX)	HPLC	40	13–207	[69]
	Miscellaneous	DON	6	SPE (SAX)	HPLC	50	13–35	[69]
	Sorghum beer	DON	120	SPE (C_18_)	ELISA	89.2	0–730 μg/L	[71]
	Maize	DON	165	SPE (amino)	UPLC-MS/MS	12	27–3842	[40]
	Maize	DON	37	NA	LC-MS/MS	100	43–435	[72]
	Soybean	DON	10	NA	LC-MS/MS	100	56–75	[72]
	Maize beer	DON	14	NA	LC-MS/MS	93	3–57	[72]
	Groundnut soup	DON	15	NA	LC-MS/MS	27	0.96–1.8	[72]
	Dagwa	DON	8	NA	LC-MS/MS	100	12–116	[72]
	Maize	NIV	37	NA	LC-MS/MS	100	3–782	[72]
	Soybean	NIV	10	NA	LC-MS/MS	90	0.3–0.5	[72]
	Maize beer	NIV	14	NA	LC-MS/MS	57	3–90	[72]
	Dagwa	NIV	8	NA	LC-MS/MS	100	51–155	[72]
	Maize	FX	37	NA	LC-MS/MS	86	<LOQ–112	[72]
	Soybean	FX	10	NA	LC-MS/MS	100	33–42	[72]
Ethiopia	Maize	DON	17	SPE (C_18_)	HPLC	29.4	50–700	[76]
	Maize	NIV	17	SPE (C_18_)	HPLC	17.7	50–210	[76]
	Barley	DON	20	NA	HPLC	35	40–110	[75]
	Sorghum	DON	33	NA	HPLC	90.9	50–2340	[75]
	Sorghum	NIV	33	NA	HPLC	9.1	50–490	[75]
	Wheat	DON	23	NA	HPLC	17.4	50–110	[75]
	Wheat	NIV	23	NA	HPLC	4.4	40 (mean)	[75]
	Sorghum	DON	70	NA	HPLC/ESI-MS/MS	2.86	nd–78.1	[77]
	Sorghum	NIV	70	NA	HPLC/ESI-MS/MS	5.71	nd–54.9	[77]
	Sorghum	DAS	70	NA	HPLC/ESI-MS/MS	35.7	nd–64.2	[77]
	Millet	DON	34	NA	HPLC/ESI-MS/MS	9.09	nd–4.1	[77]
	Millet	NIV	34	NA	HPLC/ESI-MS/MS	12.1	nd–8.1	[77]
	Millet	DAS	70	NA	HPLC/ESI-MS/MS	3.03	nd–1.4	[77]
Kenya	Wheat	DON	82	NA	ELISA	68.3	105–303	[36]
	Wheat	T2	80	NA	ELISA	76.3	20–66	[36]
	Maize beer	DON	61	NA	QuickTox Kit	23	200–360	[80]
	Wheat kernels	HT2	26	NA	LC-MS/MS	11.5	124–239	[114]
	Wheat Kernels	FX	26	NA	LC-MS/MS	15.4	14–294	[114]
	Wheat Kernels	NEO	26	NA	LC-MS/MS	11.5	20–51	[114]
	Wheat kernels	NIV	26	NA	LC-MS/MS	7.7	25–60	[114]
	Wheat kernels	DON	26	NA	LC-MS/MS	69.2	25–1310	[114]
	Kenyan Lager Beers	DON	75	SPE (C_18_)	ELISA	100	1.56–6.4 μg/L	[81]
Malawi	Maize	DON	90	NA	HPLC/ESI-MS/MS	99	nd–2328	[82]
	Maize	NIV	90	NA	HPLC/ESI-MS/MS	84	nd–2220	[82]
	Maize	DAS	90	NA	HPLC/ESI-MS/MS	37	nd–17	[82]
	Maize	FX	90	NA	HPLC/ESI-MS/MS	32	nd–664	[82]
Mozambique	Maize	DON	13	NA	HPLC/ESI-MS/MS	15.4	116–124	[68]
	Maize	NIV	13	NA	HPLC/ESI-MS/MS	30.8	20.2–45.9	[68]
	Feed	DON	10	NA	HPLC/ESI-MS/MS	50	99.1–697	[68]
	Others	DON	7	NA	HPLC/ESI-MS/MS	14	145 (median)	[68]
	Feed	NIV	10	NA	HPLC/ESI-MS/MS	20	42.7–52.7	[68]
	Others	NIV	7	NA	HPLC/ESI-MS/MS	29	76.8–113	[68]
	Maize	DON	70	NA	HPLC/ESI-MS/MS	100	11–479	[39]
	Maize	NIV	70	NA	HPLC/ESI-MS/MS	54.3	0.7–164	[39]
	Maize	DON	180	NA	LC-MS/MS	22	9.6–745.1	[115]
	Maize	DAS	180	NA	LC-MS/MS	9	1.0–51.0	[115]
	Maize	DON	136	SPE (C_18_), MultiSep	LC-MS/MS	16	99 (mean)	[88]
	Maize	HT-2	136	SPE (C_18_), MultiSep	LC-MS/MS	1	20 (mean)	[88]
	Maize	NIV	136	SPE (C_18_), MultiSep	LC-MS/MS	2	206 (mean)	[88]
	Maize	FX	136	SPE (C_18_), MultiSep	LC-MS/MS	1	154 (mean)	[88]
	Maize	DAS	136	SPE (C_18_), MultiSep	LC-MS/MS	13	3 (mean)	[88]
	Sorghum	DON	110	SPE (C_18_), MultiSep	LC-MS/MS	3	100 (mean)	[88]
	Sorghum	HT-2	110	SPE (C_18_), MultiSep	LC-MS/MS	8	20 (mean)	[88]
Nigeria	Sorghum	DAS	110	SPE (C_18_), MultiSep	LC-MS/MS	18	5 (mean)	[88]
	Millet	DON	87	SPE (C_18_), MultiSep	LC-MS/MS	13	151 (mean)	[88]
	Millet	HT-2	87	SPE (C_18_), MultiSep	LC-MS/MS	5	36 (mean)	[88]
	Millet	DAS	87	SPE (C_18_), MultiSep	LC-MS/MS	29	5 (mean)	[88]
	*Ogi*	DON	30	SPE (C_18_), MultiSep	LC-MS/MS	13	61 (mean)	[88]
	*Ogi*	HT-2	30	SPE (C_18_), MultiSep	LC-MS/MS	3	13 (mean)	[88]
	*Ogi*	NIV	30	SPE (C_18_), MultiSep	LC-MS/MS	7	148 (mean)	[88]
	*Ogi*	FX	30	SPE (C_18_), MultiSep	LC-MS/MS	7	133 (mean)	[88]
	Maize snack	NIV	8	NA	HPLC/ESI-MS/MS	25	1.8–2.5	[85]
	Rice	DON	21	NA	HPLC	23.8	11.2–112.2	[89]
Republic of Benin	Cassava flour	DAS	4	SPE (amino)	UPLC-MS/MS	100	<LOD–5	[93]
South Africa	Maize meal	DON	18	IAC	HPLC	88.9	0–960	[116]
	Wheat flour	DON	23	IAC	HPLC	69.6	0–100	[116]
	Compound feed	DON	91	SPE (SAX)	HPLC	70.3	124–2352	[96]
	Maize	DON	54	IAC	LC-MS/MS	32	4.2–675	[38]
Tanzania	Maize	DON	60	NA	UHPLC/TOFMS	63.33	68–2196	[98]
	Maize	HT2	60	NA	UHPLC/TOFMS	25	15–25	[98]
Zimbabwe	Maize	DAS	95	SPE (amino)	LC-MS/MS	1	nd–14	[100]
	Maize	DON	95	SPE (amino)	LC-MS/MS	24	nd–492	[100]
	Maize	NIV	95	SPE (amino)	LC-MS/MS	3	nd–530	[100]

Burkina Faso, others = 30 (sorghum—7, millet—3, rice—3, sesame—2, wheat—1, infant food formulations—3, mixed cuscus—3, cornflakes—2, cookies—2 and dried fruits–4); Mozambique, others = 7 (millet—2, soy—3, waste product from feed production—2); Cameroon, miscellaneous = 6 (rice, pumpkin seeds (egusi), fermented cassava flakes (garri), fermented cassava flour (nkum nkum)); nd = not detected; NA = not applicable; LOD = limit of detection; LOQ = limit of quantification; DON = deoxynivalenol; NIV = nivalenol; T2 = T-2 toxin; HT2 = HT-2 toxin; DAS = diacetoxyscirpenol; FX = Fusarenon X; NEO = neosolaniol; IAC = immunoaffinity column; SPE = solid phase extraction; SAX = strong anion exchange; ELISA = enzyme-linked immunosorbent assay; HPLC/ESI-MS/MS = liquid chromatography/electrospray ionization tandem mass spectrometry; UHPLC/TOFMS = ultra high performance liquid chromatography/time-of-flight mass spectrometry; LC-MS/MS = liquid chromatography-tandem mass spectrometry.

**Table 3:** Occurrence and contamination levels of zearalenone in food crops and products in sub-Saharan Africa since the year 2000.

Country	Sample Type	No of Samples	Sample Preparation	Technique	% Positive	Range (μg/kg)	Reference
Burkina Faso	Maize	26	NA	HPLC/ESI-MS/MS	8	11.0–15.8	[68]
	Feed	4	NA	HPLC/ESI-MS/MS	50	43.9–54.3	[68]
	Others	30	NA	HPLC/ESI-MS/MS	29	12.3–17.0	[68]
Cameroon	Maize	40	SPE (SAX)	HPLC	77.5	28–273	[69]
	Maize	18	NA	ELISA	83.3	nd–1100	[70]
	Peanut	16	SPE (SAX)	HPLC	62.5	31–186	[69]
	Bean	15	SPE (SAX)	HPLC	33.3	27–157	[69]
	Miscellaneous	6	SPE (SAX)	HPLC	16.7	67 (mean)	[69]
	Maize	37	NA	LC-MS/MS	89	0.2–309	[72]
	Groundnut	35	NA	LC-MS/MS	43	<LOQ–45	[72]
	Soybean	10	NA	LC-MS/MS	100	12–18	[72]
	Maize beer	14	NA	LC-MS/MS	86	1.6–35	[72]
	Kuru-Kuru	6	NA	LC-MS/MS	17	<LOQ	[72]
	Dagwa	8	NA	LC-MS/MS	100	6–57	[72]
Côte d’Ivoire	Maize	10	NA	ELISA	100	20–50	[73]
	Rice	10	NA	ELISA	100	50–200	[73]
	Peanut	10	NA	ELISA	100	50–200	[73]
Democratic Republic of Congo	Maize	40	SPE (SAX), (IAC)	TLC, HPLC	92.5	24–811.2	[74]
	Bean	30	SPE (SAX), (IAC)	TLC, HPLC	90	12.5–273.2	[74]
Ethiopia	Sorghum	29	NA	HPLC	6.9	19–32	[75]
	Sorghum	70	NA	HPLC/ESI-MS/MS	32.9	nd–374	[77]
	Millet	34	NA	HPLC/ESI-MS/MS	51.5	nd–459	[77]
Kenya	Wheat	82	NA	ELISA	57.3	1–96	[36]
	Wheat kernels	26	NA	LC-MS/MS	26.9	7–55	[114]
	Kenyan Lager Beers	75	SPE (C_18_)	ELISA	100	4.3–10.2 µg/L	[81]
Malawi	Maize	90	NA	HPLC/ESI-MS/MS	68	nd–2025	[82]
Mozambique	Maize	13	NA	HPLC/ESI-MS/MS	23.1	10.9–18.1	[68]
	Feed	10	NA	HPLC/ESI-MS/MS	60	11.2–28.2	[68]
	Others	7	NA	HPLC/ESI-MS/MS	43	78.8–318	[68]
	Maize	182	NA	LC-MS/MS	57	115–779	[84]
	Maize	69	NA	HPLC	5.8	2–13	[129]
	Maize	70	NA	HPLC/ESI-MS/MS	17.1	0.4–2044	[39]
Nigeria	Rice	196	NA	TLC	47.5	0–1169	[130]
	Rice	21	NA	HPLC	52.4	8.8–41.9	[89]
	Maize	136	SPE (C_18_), MultiSep	LC-MS/MS	1	65 (mean)	[88]
	Sorghum	110	SPE (C_18_), MultiSep	LC-MS/MS	1	38 (mean)	[88]
	Millet	87	SPE (C_18_), MultiSep	LC-MS/MS	14	419 (mean)	[88]
	*Ogi*	30	SPE (C_18_), MultiSep	LC-MS/MS	3	39 (mean)	[88]
Republic of Benin	Cassava flour	4	SPE (amino)	UPLC-MS/MS	100	<LOQ–12	[93]
South Africa	Traditional Beer	32	NA	TLC, HPLC	21.9	2.6–426 µg/L	[131]
	Maize	40	SPE (SAX)	HPLC	90	0–135	[37]
	Compound feed	91	SPE (SAX)	HPLC	51.6	30–610	[96]
	Maize	54	IAC	LC-MS/MS	100	56–14,990	[38]
Tanzania	Maize	60	NA	UHPLC/TOFMS	5	651–1464	[98]
Zimbabwe	Maize	95	SPE (amino)	LC-MS/MS	15	nd–369	[100]

Burkina Faso, others = 30 (sorghum—7, millet—3, rice—3, sesame—2, wheat—1, infant food formulations—3, mixed cuscus—3, cornflakes—2, cookies—2 and dried fruits–4); Mozambique, others = 7 (millet—2, soy—3, waste product from feed production—2); Cameroon, miscellaneous = 6 (rice, pumpkin seeds (egusi), fermented cassava flakes (garri), fermented cassava flour (nkum nkum); nd = not detected; NA = not applicable; LOQ = limit of quantification; IAC = immunoaffinity column; SPE = solid phase extraction; SAX = strong anion exchange; ELISA = enzyme-linked immunosorbent assay; HPLC/ESI-MS/MS = liquid chromatography/electrospray ionization tandem mass spectrometry; TLC = thin layer chromatography; UHPLC/TOFMS = ultra high performance liquid chromatography/time-of-flight mass spectrometry; LC-MS/MS = liquid chromatography-tandem mass spectrometry.

**Table 4:** Occurrence and contamination levels of emerging and modified *Fusarium* mycotoxins in food crops and processed food products in sub-Saharan Africa since the year 2000.

Country	Sample Type	Mycotoxin Type	No of Samples	Sample Preparation	Technique	% Positive	Range (μg/kg)	Reference
Burkina Faso	Others	DON-3G	30	NA	HPLC/ESI-MS/MS	7	23.6–39.7	[68]
	Maize	MON	26	NA	HPLC/ESI-MS/MS	8	413–1025	[68]
	Feed	MON	4	NA	HPLC/ESI-MS/MS	25	48(median)	[68]
	Others	MON	30	NA	HPLC/ESI-MS/MS	18	70.2–320	[68]
	Others	ENN A	30	NA	HPLC/ESI-MS/MS	21	0.3–1.4	[68]
	Feed	ENN A_1_	4	NA	HPLC/ESI-MS/MS	25	0.1 (median)	[68]
	Others	ENN A_1_	30	NA	HPLC/ESI-MS/MS	29	0.2–9.1	[68]
	Others	ENN B	30	NA	HPLC/ESI-MS/MS	29	1.2–16.4	[68]
	Maize	ENN B_1_	26	NA	HPLC/ESI-MS/MS	4	0.2 (median)	[68]
	Others	ENN B_1_	30	NA	HPLC/ESI-MS/MS	29	0.9–21.4	[68]
	Others	ENN B_2_	30	NA	HPLC/ESI-MS/MS	14	0.2–0.8	[68]
	Maize	BEA	26	NA	HPLC/ESI-MS/MS	54	0.1–5.9	[68]
	Groundnut	BEA	9	NA	HPLC/ESI-MS/MS	11	0.1 (median)	[68]
	Feed	BEA	4	NA	HPLC/ESI-MS/MS	75	25.4–31.7	[68]
Cameroon	Maize	DON-3G	37	NA	LC-MS/MS	100	<LOQ–82	[72]
	Soybean	DON-3G	10	NA	LC-MS/MS	100	<LOQ–1	[72]
	Maize beer	DON-3G	14	NA	LC-MS/MS	86	0.3–27	[72]
	Dagwa	DON-3G	8	NA	LC-MS/MS	100	4–90	[72]
	Maize beer	α-ZEL	14	NA	LC-MS/MS	86	0.6–2	[72]
	Groundnut soup	α-ZEL	15	NA	LC-MS/MS	20	0.4–0.5	[72]
	Maize beer	β-ZEL	14	NA	LC-MS/MS	93	0.03–8	[72]
	Groundnut soup	β-ZEL	15	NA	LC-MS/MS	53	0.03–0.4	[72]
	Maize beer	ZEN-4S	14	NA	LC-MS/MS	93	0.01–0.6	[72]
	Groundnut soup	ZEN-4S	15	NA	LC-MS/MS	13	0.001–0.01	[72]
	Maize	ENN A	37	NA	LC-MS/MS	38	<LOQ–0.04	[72]
	Groundnut	ENN A	35	NA	LC-MS/MS	57	<LOQ–0.1	[72]
	Groundnut soup	ENN A	15	NA	LC-MS/MS	87	<LOQ–0.1	[72]
	Groundnut	ENN A_1_	35	NA	LC-MS/MS	29	<LOQ–6	[72]
	Groundnut soup	ENN A_1_	15	NA	LC-MS/MS	100	<LOQ–0.2	[72]
	Kuru-kuru	ENN A_1_	6	NA	LC-MS/MS	87	<LOQ	[72]
	Dagwa	ENN A_1_	8	NA	LC-MS/MS	38	<LOQ–0.04	[72]
	Maize	ENN B	37	NA	LC-MS/MS	68	<LOQ–0.07	[72]
	Groundnut	ENN B	35	NA	LC-MS/MS	91	<LOQ–0.6	[72]
	Soybean	ENN B	10	NA	LC-MS/MS	10	<LOQ	[72]
	Maize beer	ENN B	14	NA	LC-MS/MS	50	0.004–0.02	[72]
	Groundnut soup	ENN B	15	NA	LC-MS/MS	100	<LOQ–0.2	[72]
	Kuru-Kuru	ENN B	6	NA	LC-MS/MS	100	0.02–0.03	[72]
	Dagwa	ENN B	8	NA	LC-MS/MS	88	<LOQ–0.1	[72]
	Maize	ENN B_1_	37	NA	LC-MS/MS	89	<LOQ–1	[72]
	Groundnut	ENN B_1_	35	NA	LC-MS/MS	91	0.02–5	[72]
	Soybean	ENN B_1_	10	NA	LC-MS/MS	50	0.01–0.04	[72]
	Maize beer	ENN B_1_	14	NA	LC-MS/MS	57	0.01–0.4	[72]
	Groundnut soup	ENN B_1_	15	NA	LC-MS/MS	93	0.01–0.3	[72]
	Kuru-Kuru	ENN B_1_	6	NA	LC-MS/MS	100	0.2–0.4	[72]
	Dagwa	ENN B_1_	8	NA	LC-MS/MS	100	0.01–0.8	[72]
	Maize	BEA	37	NA	LC-MS/MS	100	0.3–93	[72]
	Groundnut	BEA	35	NA	LC-MS/MS	100	0.2–12	[72]
	Soybean	BEA	10	NA	LC-MS/MS	100	12–19	[72]
	Maize beer	BEA	14	NA	LC-MS/MS	93	2–11	[72]
	Groundnut soup	BEA	15	NA	LC-MS/MS	100	0.04–1	[72]
	Kuru-Kuru	BEA	6	NA	LC-MS/MS	100	0.6–0.9	[72]
	Dagwa	BEA	8	NA	LC-MS/MS	100	3.4–31	[72]
	Sorghum	DON-3G	70	NA	HPLC/ESI-MS/MS	7.14	nd–4.7	[77]
	Sorghum	ZEN-4S	70	NA	HPLC/ESI-MS/MS	5.71	nd–2.4	[77]
	Sorghum	α-ZEL	70	NA	HPLC/ESI-MS/MS	7.14	nd–8.3	[77]
	Sorghum	β-ZEL	70	NA	HPLC/ESI-MS/MS	8.57	nd–23.8	[77]
	Sorghum	ENN B	70	NA	HPLC/ESI-MS/MS	47.1	nd–0.7	[77]
	Sorghum	ENN B_1_	70	NA	HPLC/ESI-MS/MS	44.3	nd–2.7	[77]
	Sorghum	ENN A_1_	70	NA	HPLC/ESI-MS/MS	38.6	nd–4.0	[77]
	Sorghum	ENN A	70	NA	HPLC/ESI-MS/MS	21.4	nd–0.8	[77]
	Sorghum	BEA	70	NA	HPLC/ESI-MS/MS	71.4	nd–290	[77]
	Sorghum	MON	70	NA	HPLC/ESI-MS/MS	97.1	nd–316	[77]
	Sorghum	FA	70	NA	HPLC/ESI-MS/MS	7.14	nd–239	[77]
	Millet	α-ZEL	34	NA	HPLC/ESI-MS/MS	21.2	nd–6.5	[77]
	Millet	β-ZEL	34	NA	HPLC/ESI-MS/MS	24.2	nd–4.4	[77]
Ethiopia	Millet	ZEN-4S	34	NA	HPLC/ESI-MS/MS	42.4	nd–13.9	[77]
	Millet	MAS	34	NA	HPLC/ESI-MS/MS	3.03	nd–2.7	[77]
	Millet	BEA	34	NA	HPLC/ESI-MS/MS	100	85.6 (maximum)	[77]
	Millet	ENN B	34	NA	HPLC/ESI-MS/MS	69.7	nd–1.8	[77]
	Millet	ENN B_1_	34	NA	HPLC/ESI-MS/MS	78.8	nd–5.3	[77]
	Millet	ENN A_1_	34	NA	HPLC/ESI-MS/MS	57.6	nd–3.0	[77]
	Millet	ENN A	34	NA	HPLC/ESI-MS/MS	21.2	nd–0.4	[77]
	Millet	FA	34	NA	HPLC/ESI-MS/MS	18.2	nd–241	[77]
	Millet	MON	34	NA	HPLC/ESI-MS/MS	57.6	nd–46.9	[77]
Kenya	Wheat Kernels	3-ADON	26	NA	LC-MS/MS	34.6	80–1703	[114]
	Wheat Kernels	15-MAS	26	NA	LC-MS/MS	7.7	42–107	[114]
	Wheat Kernels	MON	26	NA	LC-MS/MS	7.7	5–17	[114]
	Wheat Kernels	ENN B	26	NA	LC-MS/MS	50	2–256	[114]
	Wheat Kernels	BEA	26	NA	LC-MS/MS	7.7	13–15	[114]
Malawi	Maize	BEA	90	NA	HPLC/ESI-MS/MS	99	nd–415	[82]
	Maize	MON	90	NA	HPLC/ESI-MS/MS	94	nd–1624	[82]
	Maize	FA	90	NA	HPLC/ESI-MS/MS	72	nd–1020	[82]
	Maize	FUS	90	NA	HPLC/ESI-MS/MS	70	nd–2056	[82]
	Maize	HYD FB_1_	90	NA	HPLC/ESI-MS/MS	61	nd–30	[82]
	Maize	ZEN-4S	90	NA	HPLC/ESI-MS/MS	50	nd–80	[82]
	Maize	DON-3G	90	NA	HPLC/ESI-MS/MS	28	nd–268	[82]
	Maize	ENN	90	NA	HPLC/ESI-MS/MS	20	nd–9.5	[82]
	Maize	β-ZEL	90	NA	HPLC/ESI-MS/MS	18	nd–124	[82]
	Maize	α-ZEL	90	NA	HPLC/ESI-MS/MS	7	nd–56	[82]
	Maize	T-2T	90	NA	HPLC/ESI-MS/MS	3	nd–123	[82]
	Maize	MON	13	NA	HPLC/ESI-MS/MS	54	98–1305	[68]
	Feed	MON	10	NA	HPLC/ESI-MS/MS	80	61.0–1601	[68]
	Others	MON	7	NA	HPLC/ESI-MS/MS	57	46.8–1923	[68]
	Feed	ENN A	10	NA	HPLC/ESI-MS/MS	40	0.6–7.9	[68]
	Others	ENN A	7	NA	HPLC/ESI-MS/MS	29	0.2–2.0	[68]
	Maize	ENN A_1_	13	NA	HPLC/ESI-MS/MS	15	0.1–0.1	[68]
	Feed	ENN A_1_	10	NA	HPLC/ESI-MS/MS	40	3.4–43.9	[68]
	Others	ENN A_1_	7	NA	HPLC/ESI-MS/MS	29	0.2–4.1	[68]
Mozambique	Feed	ENN B	10	NA	HPLC/ESI-MS/MS	40	2.2–114	[68]
	Others	ENN B	7	NA	HPLC/ESI-MS/MS	14	0.9 (median)	[68]
	Maize	ENN B_1_	13	NA	HPLC/ESI-MS/MS	8	0.1 (median)	[68]
	Groundnut	ENN B_1_	23	NA	HPLC/ESI-MS/MS	5	0.3 (median)	[68]
	Feed	ENN B_1_	10	NA	HPLC/ESI-MS/MS	70	0.1–94.4	[68]
	Others	ENN B_1_	7	NA	HPLC/ESI-MS/MS	14	4.1 (median)	[68]
	Feed	ENN B_2_	10	NA	HPLC/ESI-MS/MS	30	0.9–9.1	[68]
	Maize	BEA	13	NA	HPLC/ESI-MS/MS	85	0.1–35.6	[68]
	Groundnut	BEA	23	NA	HPLC/ESI-MS/MS	73	0.1–24.0	[68]
	Feed	BEA	10	NA	HPLC/ESI-MS/MS	100	3.3–418	[68]
	Others	BEA	7	NA	HPLC/ESI-MS/MS	100	3.5–486	[68]
Nigeria	Maize	α-ZEL	182	NA	LC-MS/MS	14	32–181	[84]
	Stored Maize	α-ZEL	70	NA	HPLC/ESI-MS/MS	1.4	17 (mean)	[39]
	Stored Maize	β-ZEL	70	NA	HPLC/ESI-MS/MS	1.4	13 (mean)	[39]
	Maize	3-ADON	180	NA	LC-MS/MS	17.2	0.7–72	[115]
	Stored Maize	BEA	70	NA	HPLC/ESI-MS/MS	78.6	0.1–120	[39]
	Groundnut Snack	BEA	10	NA	HPLC/ESI-MS/MS	60	2–84	[85]
	Maize Snack	BEA	8	NA	HPLC/ESI-MS/MS	100	0.6–5.2	[85]
	Groundnut/Maize snack	BEA	2	NA	HPLC/ESI-MS/MS	100	1.8–1.9	[85]
	Maize Snack	ENN B_2_	8	NA	HPLC/ESI-MS/MS	12.5	0.1(mean)	[85]
	Stored Maize	FUS	70	NA	HPLC/ESI-MS/MS	4.3	57.4–263	[39]
	Stored Maize	DON-3G	70	NA	HPLC/ESI-MS/MS	10	0.1–76	[39]
	Stored Maize	HYD FB_1_	70	NA	HPLC/ESI-MS/MS	52.9	0.4–135	[39]
	Stored Maize	MON	70	NA	HPLC/ESI-MS/MS	77.1	0.8–899	[39]
	Maize	15-MAS	32	NA	GC-MS	3.1	4 (mean)	[129]
	Maize	T-2T	32	NA	GC-MS	6.3	73–280	[129]
	Maize	ZEN-14G	136	SPE (C_18_), MultiSep	LC-MS/MS	9	21 (mean)	[88]
	Maize	α-ZEL	136	SPE (C_18_), MultiSep	LC-MS/MS	1	20 (mean)	[88]
	Maize	β-ZEL	136	SPE (C_18_), MultiSep	LC-MS/MS	2	20 (mean)	[88]
	Sorghum	15-ADON	110	SPE (C_18_), MultiSep	LC-MS/MS	2	39 (mean)	[88]
	Sorghum	DON-3G	110	SPE (C_18_), MultiSep	LC-MS/MS	23	24 (mean)	[88]
	Sorghum	ZEN-14G	110	SPE (C_18_), MultiSep	LC-MS/MS	3	19 (mean)	[88]
	Sorghum	α-ZEL	110	SPE (C_18_), MultiSep	LC-MS/MS	3	33 (mean)	[88]
	Sorghum	β-ZEL	110	SPE (C_18_), MultiSep	LC-MS/MS	1	21 (mean)	[88]
	Millet	15-ADON	87	SPE (C_18_), MultiSep	LC-MS/MS	1	11 (mean)	[88]
	Millet	ZEN-14G	87	SPE (C_18_), MultiSep	LC-MS/MS	6	23 (mean)	[88]
	Millet	β-ZEL	87	SPE (C_18_), MultiSep	LC-MS/MS	1	39 (mean)	[88]
	*Ogi*	15-ADON	30	SPE (C_18_), MultiSep	LC-MS/MS	3	60 (mean)	[88]
	*Ogi*	DON-3G	30	SPE (C_18_), MultiSep	LC-MS/MS	17	30 (mean)	[88]
	*Ogi*	ZEN-14G	30	SPE (C_18_), MultiSep	LC-MS/MS	3	31 (mean)	[88]
	*Ogi*	α-ZEL	30	SPE (C_18_), MultiSep	LC-MS/MS	7	20 (mean)	[88]
	*Ogi*	β-ZEL	30	SPE (C_18_), MultiSep	LC-MS/MS	10	19 (mean)	[88]
	Peanut cake	BEA	29	NA	HPLC/ESI-MS/MS	100	0.05–3.4	[175]
	Poultry feed	BEA	58	NA	HPLC/ESI-MS/MS	100	3–39	[90]
	Poultry feed	ENN A	58	NA	HPLC/ESI-MS/MS	74	0.3–15	[90]
	Poultry feed	ENN A_1_	58	NA	HPLC/ESI-MS/MS	79	0.5–101	[90]
	Poultry feed	ENN B	58	NA	HPLC/ESI-MS/MS	91	0.1–141	[90]
	Poultry feed	ENN B_1_	58	NA	HPLC/ESI-MS/MS	81	1–182	[90]
	Poultry feed	ENN B_2_	58	NA	HPLC/ESI-MS/MS	22	1–8	[90]
	Poultry feed	ENN B_3_	58	NA	HPLC/ESI-MS/MS	2	0.007 (mean)	[90]
	Poultry feed	HYD FB_1_	58	NA	HPLC/ESI-MS/MS	16	2–11	[90]
Zimbabwe	Maize	15-ADON	95	SPE (amino)	LC-MS/MS	4	nd–105	[100]

Burkina Faso, others = 30 (sorghum—7, millet—3, rice—3, sesame—2, wheat—1, infant food formulations—3, mixed cuscus—3, cornflakes—2, cookies—2 and dried fruits–4); Mozambique, others = 7 (millet—2, soy—3, waste product from feed production—2); nd = not detected; NA = not applicable; LOQ = limit of quantification; BEA = beauvericin; ENN = enniatin; MON = moniliformin; FUS = fusaproliferin; 15-MAS = 15-monoacetoxyscirpenol; FA = fusaric acid; 3-ADON = 3-acetyldeoxynivalenol; 15-ADON = 15-acetyldeoxynivalenol; α-ZEL = α-zearalenol; β-ZEL = β-zearalenol; DON-3G = deoxynivalenol-3-glucoside; HYD FB_1_ = Hydrolysed FB_1_; ZEN-4S = zearalenone-4-sulfate; T-2T = T2 Tetraol; SPE = solid phase extraction; GC-MS = gas chromatography/mass spectrometry; HPLC/ESI-MS/MS = liquid chromatography/electrospray ionization tandem mass spectrometry; LC-MS/MS = liquid chromatography-tandem mass spectrometry.

## Abbreviation

The following abbreviations are used in this manuscript:

3-ADON	3-acetyldeoxynivalenol
15-ADON	15-acetyldeoxynivalenol
15-MAS	15-monoacetoxyscirpenol
α-ZEL	α-zearalenol
β-ZEL	β-zearalenol
BEA	beauvericin
CAC	Codex Alimentarius Commission
DAS	diacetoxyscirpenol
DOM	de-epoxy deoxynivalenol
DON	deoxynivalenol
DON-3G	deoxynivalenol-3-glucoside
ELISA	enzyme-linked immunosorbent assay
ENN	enniatin
EU	European Union
FA	fusaric acid
FAO	Food and Agriculture Organization
FB	fumonisin
FP	fusaproliferin
FX	fusarenon X
FUS	fusaproliferin
GC-MS	gas chromatography/mass spectrometry
GAP	good agricultural practices
GMP	good manufacturing practices
HACCP	hazard analysis and critical control points
HPLC	high-performance liquid chromatography
HPLC/ESI-MS/MS	liquid chromatography/electrospray ionization tandem mass spectrometry
HR-MS	high-resolution mass spectrometry
HT2	HT-2 toxin
HT-2G	HT-2 glucoside
HYD	hydrolyzed FB_1_
IAC	immunoaffinity column,
IARC	International Agency for Research on Cancer
LC-MS/MS	liquid chromatography-tandem mass spectrometry
LFIA	lateral flow immunoassay
LOQ	limit of quantification
MON	moniliformin
MTT	3-(4,5-dimethylthiazol-2-yl)-2,5-diphenyltetrazolium bromide
NA	not applicable
nd	not detected
NEO	neosolaniol
NIV	nivalenol
SAX	strong anion exchange
SPE	solid phase extraction
SSA	sub-Saharan Africa
T2	T-2 toxin
T-2G	T-2 glucoside
T-2T	T2 tetraol
TH	trichothecenes
TLC	thin layer chromatography
UHPLC/TOFMS	ultra-high performance liquid chromatography/time-of-flight mass spectrometry
ZEN	zearalenone
ZEN-4S	zearalenone-4-sulfate
ZEN-14G	zearalenone-14-glucoside
ZEN-14S	zearalenone-14-sulfate
